# Is H19 RNA a Useful Marker of Acromegaly and Its Complications? A Preliminary Study

**DOI:** 10.3390/biomedicines11041211

**Published:** 2023-04-19

**Authors:** Małgorzata Rolla, Aleksandra Jawiarczyk-Przybyłowska, Katarzyna Kolačkov, Agnieszka Zembska, Marek Bolanowski

**Affiliations:** Department of Endocrinology, Diabetes and Isotope Therapy, Wroclaw Medical University, Wybrzeże Pasteura 4, 50-367 Wrocław, Polandmarek.bolanowski@umw.edu.pl (M.B.)

**Keywords:** H19, acromegaly, lncRNA

## Abstract

Acromegaly is a rare endocrine disorder caused by somatotroph pituitary adenoma. Besides its typical symptoms, it contributes to the development of cardiovascular, metabolic, and bone comorbidities. H19 RNA is a long non-coding RNA and it is suspected to be involved in tumorigenesis, cancer progression, and metastasis. H19 RNA is a novel biomarker for the diagnosis and monitoring of neoplasms. Moreover, there might be an association between H19 and cardiovascular and metabolic diseases. We enrolled 32 acromegaly patients and 25 controls. We investigated whether whole blood H19 RNA expression is associated with the diagnosis of acromegaly. Correlations between H19 and tumour dimension, invasiveness, and biochemical and hormonal parameters were evaluated. We analysed the coincidence of acromegaly comorbidities with H19 RNA expression. In the results, we did not observe a statistically significant difference in H19 RNA expression between acromegaly patients and the controls. There were no correlations between H19 and the adenoma size and infiltration and patients’ biochemical and hormonal statuses. In the acromegaly group, hypertension, goitre, and cholelithiasis were observed more frequently. The diagnosis of acromegaly was a factor contributing to the occurrence of dyslipidaemia, goitre, and cholelithiasis. We found an association between H19 and cholelithiasis in acromegaly patients. To conclude, H19 RNA expression is not a relevant marker for diagnosis and monitoring of acromegaly patients. There is a higher risk of hypertension, goitre, and cholelithiasis related to acromegaly. Cholelithiasis is associated with a higher H19 RNA expression.

## 1. Introduction

Acromegaly is a disease caused by excess production of growth hormone (GH), mainly due to pituitary somatotroph adenoma [1]. Pituitary adenomas are benign tumours originating from adenohypophysis cells [2,3]. Regardless of the lack of malignant potential, some of them can be locally invasive or have high cell proliferative activity [2], leading to nonradical treatment or a higher risk of recurrence. Somatotroph adenomas are mainly macroadenomas, defined as tumours exceeding 10 mm in the maximal dimension [4]. Due to their size, some of them infiltrate adjacent structures, such as optic chiasm, sphenoid and cavernous sinuses, and bone. The clinical manifestation of acromegaly results from GH and insulin-like growth factor I (IGF-I) overproduction and tumour mass effect. Acromegaly patients present facial and acral enlargement, joint pain, headaches, and bitemporal hemianopsia. Systemic complications, including bone and joint, cardiovascular, respiratory, and metabolic comorbidities, are the major problems affecting patient quality of life and life expectancy [5,6,7]. Multidisciplinary care is recommended for effective management. Furthermore, a delay in the diagnosis of acromegaly is a common problem due to the variety of symptoms and rare prevalence of the disease [8]. Therefore, novel biomarkers could be helpful tools in diagnosis and monitoring of acromegaly patients.

Recently, there has been a wealth of research presenting hypotheses about the pathogenesis of neoplasms’ origin and processes contributing to their progression. The search for novel biomarkers that could be useful in early stage diagnosis, which could optimize management and patient monitoring, is an important matter. Non-coding RNAs are a huge part of the human genome and lack an open reading frame encoding a functional protein [9]. Long non-coding RNAs (lncRNAs) are nucleic acids over 200 nucleotides long. They have a role in the regulation of transcription, translation, and epigenetic processes [10,11]. Among them, H19 lncRNA is the earliest investigated and was first described by Pachnis et al. in 1984 [12]. Human H19 is a 2.3-kb RNA molecule encoded by the H19 gene located on chromosome 11p15.5 [13]. Initially, H19 RNA was reported to be related to embryogenesis, with its high expression in the foetal heart, muscle, and liver. It is also downregulated after birth [12,14]. Further investigations revealed its potential role in tumorigenesis. The contribution of the different mechanisms of H19 to neoplasm origins and progression has been investigated. The H19 gene is expressed only from the maternal allele in the imprinting process [15]. The loss of imprinting and alterations in the methylation pattern of promoter sequences were initially evaluated processes in colorectal, lung, and oesophageal cancers [16,17]. Moreover, research revealed that H19 promotes cell migration, invasion, and angiogenesis and inhibits apoptosis [18]. Further investigations determined the role of H19 as an oncogene [19,20,21,22] or, on the contrary, as a tumour suppressor [23,24]. Up to now, the association between H19 and different types of cancers has been revealed for, i.e., breast [25,26], lung [17,22,27], oesophageal [28], colorectal [29,30], and bladder [31,32] cancers. Due to the variable expression of H19 in different types of tumours, its role as a tumour marker was speculated. Moreover, the downregulation of H19 expression in postoperative patients was revealed in some types of cancers [32,33,34]; hence, the monitoring function of H19 was revealed.

Evidence that H19 lncRNA contributes to tumorigenesis has been shown in many types of malignancies. However, papers concerning H19 and pituitary tumours are limited. The majority of previous studies has been performed on cell lines or animal models. Only one research work has characterized the role of H19 in a large group of patients with pituitary adenomas [35]. There is a lack of studies focused on patients diagnosed with acromegaly.

In some studies, the inhibitory role of H19 in pituitary adenomas has been suggested [36]. The downregulation of H19 RNA in pituitary adenoma tissues compared with the normal pituitary glands has been revealed [37]. H19 has been reported to suppress tumour growth by inhibiting the phosphorylation of 4E-binding protein 1 (4E-BP1). Moreover, the expression of H19 in plasma exosomes of patients with pituitary adenomas was significantly lower than in healthy controls [35]. Additionally, the suppressive influence of H19 on tumour cell proliferation and the negative correlation between H19 and pituitary tumour volume has been shown [35,37]. In recent studies, its synergistic effect with cabergoline on inhibiting tumour growth has been demonstrated for prolactinomas [35,38]. To the best of our knowledge, the interaction between H19 and somatostatin analogues has not been explored.

H19 has also been determined to play a role in the pathogenesis of other diseases. This has been shown for central nervous system disorders, such as epilepsy, Parkinson’s, and Alzheimer’s diseases [36]. Additionally, H19s associations with osteoarthritis [39], osteoporosis [40], cardiovascular [41,42], and metabolic disorders [43,44] have been revealed.

lncH19 RNA presents itself as a novel biomarker of tumorigenesis, and since the papers focusing on GH-secreting adenomas are limited, the aim of the present study was to investigate whether lncH19 RNA expression could be useful in acromegaly patients. Additionally, we evaluated whether H19 expression depends on the clinical characteristics of the patients, their treatment, tumour invasiveness, or prevalence of acromegaly comorbidities to explore if H19 RNA could be applied in prognostics and monitoring of the status of the disease and its complications.

## 2. Materials and Methods

The study group consisted of 32 patients with acromegaly (24 women and 8 men; mean age 51.97 ± 13.93 yrs). Twenty-five healthy patients were enrolled in the control group (16 women and 9 men; mean age 41.08 ± 13.79 yrs). The inclusion criterion for the study group was the diagnosis of acromegaly, either currently or in the past, defined as elevated IGF-I levels and unsuppressed GH in OGTT, according to the current recommendations of the Endocrine Society and Polish Society of Endocrinology [1,45]. The exclusion criteria for the control group were pituitary tumours confirmed by MRI or the history of other neoplasms. All the participants were recruited from the Department of Endocrinology, Diabetes and Isotope Therapy, Wroclaw Medical University. The bioethics committee of Wroclaw Medical University approved the protocol of the study (no. KB-36/2020). All subjects signed informed consent forms following the Declaration of Helsinki.

Medical histories were taken from all the participants, including current and past treatment and coexisting diseases. These data were used to categorize the patients into subgroups of the acromegaly de novo, pharmacologically treated or successfully operated patients. We recorded patients’ weight and height and calculated their body mass indexes (BMI). Fasting venous blood samples were collected from all the participants. IGF-I levels were expressed in relation to the upper limit of normal (ULN) for age (patient’s IGF-I concentration divided by IGF-I upper reference range limit matched for age). A pituitary MRI examination (1.5 Tesla) was performed on all the participants. MRI was not repeated if it had been performed in the past 6 months. According to these results, we classified the pituitary tumours as macro- (if at least one dimension was ≥10 mm) or microadenomas and analysed their extrasellar expansion.

The GH, IGF-I, thyrotropic hormone (TSH), free thyroxine (fT4), prolactin (PRL), follicle-stimulation hormone (FSH), luteinizing hormone (LSH), oestradiol (E2), total testosterone (T), dehydroepiandrosterone sulphate (DHEAS), adrenocorticotropic hormone (ACTH), and cortisol concentrations were assayed using a chemiluminescence immunoassay. Vitamin D levels were also measured by chemiluminescence immunoassays. Serum calcium was assayed using a colorimetric assay with a reference range of 8.4–10.2 mg/dL. Glucose levels were measured using the hexokinase method. The total cholesterol was assayed using a routine enzymatic method with oxidase, esterase, and peroxidase; low-density lipoprotein (LDL) was estimated by the Friedewald Equation; high-density lipoprotein (HDL) was measured by the direct assay precipitation method using a combination of polymer polyanions; and triglycerides were assayed using a routine enzymatic method.

Five millilitres of whole blood samples from all the participants were collected in PAXgene Blood RNA Tubes (Qiagen GmbH, Hilden, Germany) and stored at −70 °C until further processing. The total RNA from collected samples was extracted according to the protocol of PAXgene Blood miRNA (Qiagen GmbH, Hilden, Germany).

The concentration and quality of the purified RNA were evaluated by measuring the absorbance at A260 and A280 nm in a NanoPhotometer Touch UV/VIS (Implen GmbH, München, Germany). The recommended optimal and equal amount of total RNA was reverse transcribed using a RT2 First Strand Kit (Qiagen GmbH, Hilden, Germany).

Two genes, GAPDH and ACTB, were used as the reference genes for the normalization of lncH19 expression in the presence of XpressRef Universal Total RNA control (Qiagen GmbH, Hilden, Germany).

Reactions as controls for cDNA contaminations, NTC (no template control) and NRT (no reverse transcriptase control), were also prepared for all the experimental samples.

A quantitative real-time PCR reaction was performed by using a RT2 qPCR FAST SYBR Green according to the protocol provided by the manufacturer (Qiagen GmbH, Hilden, Germany).

Analyses of gene expression were performed on a Corbett Rotor-Gene Real-Time cycler (Qiagen GmbH, Hilden, Germany). All experiments were run in duplicate. The Ct (cycle threshold) was averaged for each sample. The relative gene expression of lncRNA H19 was evaluated by using the 2-ΔΔCt method.

Statistical analyses were performed using Statistica 13.3 (TIBCO Software, Inc., Palo Alto, CA, USA). Variables were presented as means with standard deviations (SD) and medians with interquartile ranges (IQR). A Shapiro–Wilk test was used to determine the normality of the variables. A student’s t-test was applied when normality was indicated. Mann–Whitney and Kruskal–Wallis tests were used for data not normally distributed. Proportional differences were tested using Chi-square Pearson’s and Fisher’s exact tests. Correlations were determined using Spearman’s rank correlation test. Logistic regression was used to test if an interaction between acromegaly and age influenced the occurrence of comorbidities. A robust nonparametric ANCOVA in R using the WRS2 package [46] was used to analyse the association between age and acromegaly diagnosis with H19 expression. *p* values of <0.05 were considered statistically significant.

## 3. Results

The general characteristics of the acromegaly patients and the controls are presented in Table 1. The mean weight and median body mass index (BMI) were higher compared to the control group (*p* = 0.018 and 0.001, respectively), whereas the mean height was lower in the acromegaly group (*p* = 0.045).

The study group included five patients with acromegaly de novo, fifteen patients during treatment with somatostatin analogues (SSA), and a group of twelve successfully operated patients. Twenty patients underwent one transsphenoidal surgery, three patients were operated on twice, and one patient was operated on three times with this method. One patient had transcranial reoperation. Radiotherapy was performed in four cases. Neither surgery nor radiation of the pituitary gland region were performed in the control group. Among patients who belonged to de novo or SSA-treated subgroups, eleven (55%) patients had macroadenomas (mean maximal dimension 18.7 mm). In the acromegaly de novo group, three of five patients had macroadenomas (mean maximal dimension 17.3 mm). Extrasellar invasion was detected in eight patients (40%). Pituitary insufficiency was diagnosed in fourteen acromegaly patients and six controls. The most frequent impairment in the acromegaly group was secondary hypogonadism. All patients received adequate substitution of hormones.

### 3.1. H19 Expression

There was no statistically significant difference in H19 expression between women and men. A significant positive correlation between H19 expression and age was obtained in the acromegaly group (*p* = 0.025; r = 0.396 for −ΔΔCt H19-BACT), which was not observed for the controls. On account of the age difference between acromegaly and control groups, a nonparametric ANCOVA in R with WRS2 package was used. We found no difference in H19 expression between acromegaly patients and the control group (the interaction between age and the occurrence of acromegaly did not influence variables in H19 expression; the results are illustrated in Table 2).

We found no difference in H19 expression between acromegaly patients and the control group (Table S1). Additionally, H19 expression did not correspond with the hormonal statuses of the patients. No significant correlations between H19 and GH, IGF-I, or IGF-I x ULN concentrations or other hormones levels were obtained. We did not observe a variable expression of H19 between the groups of patients without operation or radiotherapy, successfully cured patients, and the controls (Table S2). We also did not observe statistically significant differences in the expression of H19 between successfully treated patients compared to unoperated patients or patients with ineffective surgery, as well as in comparison to the control group (Table S3).

Patients with active acromegaly (de novo or uncontrolled during SSA therapy) did not vary in H19 expression from radically operated or controlled subjects (Table S4). Additionally, SSA-treated patients did not differ in H19 expression between de novo patients and the controls (Table S5). Patients treated with radiotherapy did not present variable H19 expression from de novo patients and the control group (Table S6).

We found no correlation between maximal tumour dimension and H19 expression. The H19 expression was not associated with extrasellar invasion. No variations in H19 expression were observed between patients with pituitary insufficiency among all participants nor between acromegaly and control groups.

No significant correlations were found between H19 expression and biochemical results (glucose, total cholesterol, HDL, LDL, triglycerides, calcium, and vitamin D) in acromegaly patients.

### 3.2. Comorbidities

The occurrence of comorbidities is presented in Table 3. Goitre, dyslipidaemia, and hypertension were the most frequent comorbid diseases in the acromegaly group. They were observed in at least half of the group. Acromegaly patients suffered from hypertension, goitre, and cholelithiasis more frequently compared to the control group. The difference for dyslipidaemia was on the verge of statistical significance.

We analysed which factor—a diagnosis of acromegaly or age—contributed to those variables. According to the logistic regression, we found that age, not acromegaly, was a significant predictor for the occurrence of hypertension (*p* = 0.000; OR = 1.152; 95%CI 1.063–1.200; R2 = 0.556). For dyslipidaemia, we found that acromegaly was a significant predictor (*p* = 0.024; OR = 89.738; 95%CI 1.102–7307.195; R2 = 0.203), independent from age. A similar result was achieved for goitre (*p* = 0.027; OR = 2328.933; 95%CI 2.366–2,292,682.85; R2 = 0.458). Cholelithiasis was observed only in acromegaly patients. Age was not associated with the prevalence of this complication (*p* = 0.880).

Patients with cholelithiasis had higher H19 expressions compared to patients without cholelithiasis and the controls. This result was on the verge of statistical significance in the Kruskal–Wallis test (*p* = 0.039) and in post hoc tests (acromegaly with cholelithiasis vs. acromegaly without cholelithiasis *p* = 0.068; acromegaly with cholelithiasis vs. controls: *p* = 0.058). We did not observe any significant variation in H19 expression depending on the occurrence of hypertension, dyslipidaemia, diabetes, prediabetes, osteoporosis, goitre, nephrolithiasis, or colon polyps.

## 4. Discussion

In our research, we did not find a significant variation in H19 expression between patients with somatotroph adenomas and healthy controls. Our results do not correspond with previous studies, where the inhibitory role of H19 in pituitary adenomas was suggested [35]. An analysis of H19 expression in peripheral whole blood, not pituitary gland tissues or plasma exosomes, might affect the results of our experiment. The majority of previous studies analysed the tissue expression of H19. Upregulated tissue-specific expression of H19 was found in, e.g., breast [26], colorectal [29], and oesophageal [28] cancers. On the contrary, in one of the studies, the diagnostic value of tissue H19 was not presented for breast cancer [47]. For pituitary glands, Wu et al. revealed the downregulation of H19 in tissue samples of pituitary tumours compared to non-neoplastic gland specimens [37]. The plasma expression of H19 was significantly higher, and hence was presented as a useful marker in non-small cell lung [27] and gastric [48] cancers. Additionally, peripheral blood H19 was presented as a useful diagnostic tool in sepsis [49], myocardial infarction [42], osteoarthritis [39], and epilepsy [50]. Exosomal H19 expression has been proven as a marker of breast and bladder cancers [32,34]. Regarding pituitary tumours, Zhang et al. proved a reduced H19 expression in exosomes derived from the blood of patients with all types of pituitary adenomas [35]. For breast cancer, both tumour tissue and plasma H19 expressions were increased compared to healthy controls [33]. Additionally, in diabetes mellitus patients, there was no significant difference between serum and exosomal expression of H19 [51]. There is a lack of studies comparing tissue or exosomal H19 expression with the whole blood on the subject of pituitary adenomas.

We did not find any association between H19 and maximal tumour dimension. In contrast to the above reports, a significantly higher H19 expression was reported in invasive GH tumour samples in comparison to non-invasive ones [52]. However, in our study, we did not observe any differences in H19 expression between patients with invasive and non-invasive adenomas. We also did not observe a distinction in H19 expression between patients de novo and pharmacologically treated. The impacts of dopamine agonists have been evaluated in previous papers [35,38] and there is no information concerning somatostatin analogues to compare. In our study, we did not observe a variation in H19 expression between the patients after surgery or radiotherapy and the untreated group. To the best of our knowledge, there are no research works analyzing H19 expression in terms of surgical or radiation treatment of pituitary adenoma patients.

Moreover, the lack of a difference in H19 expression between women and men agrees with previous studies [27,39]. We observed a positive correlation between H19 expression and age in acromegaly patients, which is in contrast to some studies that presented no correlation between H19 expression and age [27,53].

Acromegaly patients are at a high risk of cardiovascular diseases, metabolic disorders, and endocrine comorbidities [6]. Excesses of GH and IGF-I lead to remodelling of tissues and organs, including the heart and vessels; increases in lipolysis and insulin resistance; disruption of bone turnover; and predisposition of the patient to thyroid enlargement and colon polyps [54]. The distribution of comorbidities in acromegaly patients detected in this research is similar to the results that we presented in our previous observational study [55]. Dyslipidaemia, goitre, and hypertension were the complications with the highest frequency. Hypertension and dyslipidaemia are also diseases of affluence, with a high prevalence in the general population. However, we revealed that acromegaly was an independent factor contributing to the occurrence of dyslipidaemia and high triglycerides level. No statistical correlation was found between lipid profile and H19. Similar results were presented by Safaei et al. [42]. The occurrence of acromegaly was not a prognostic factor for hypertension, diabetes, or prediabetes, whereas those complications were also frequent in the group of acromegaly. High occurrences of hypertension and metabolic disorders enhance the risk of cardiovascular events. The occurrence of goitre was significantly higher in the study group compared to the controls, and the diagnosis of acromegaly was an independent factor influencing this result. The extremely high OR and an upper limit of 95% confidence could be the result of a small sample size. In the literature, a higher predisposition to goitre, autonomous function, and even thyroid cancer was described [54,56]. Additionally, we observed a higher incidence of cholelithiasis in acromegaly patients. Acromegaly by itself predisposes the patient to this complication, but treatment with somatostatin analogues may enhance the risk [57,58]. Moreover, patients with the co-occurrence of cholelithiasis had higher H19 expressions in comparison to patients without cholelithiasis and the controls. In the literature, there is a lack of information about the association between H19 and cholelithiasis, so this result is a novel finding. Fawzy et al. found an increased H19 expression in the serum of patients with type 2 diabetes [44], whereas contrasting results and a decreased H19 expression were reported by Alfaifi et al. [43]. In our report, we did not observe significant differences in H19 expressions between diabetic and non-diabetic patients. Furthermore, some contrasting results were reported for the H19 expression in postmenopausal osteoporosis patients. In Xiaoling et al.’s research, a significant decrease in H19 was shown [59], whereas Li et al. found significantly a higher expression in this group [60]. We did not obtain a different H19 expression in patients who suffered from osteoporosis. Overexpression of H19 in colorectal cancer has also been reported [61,62]. In our patient group, only benign colon polyps were observed. Patients with colon polyps did not present different H19 expressions.

## 5. Conclusions

To the best of our knowledge, this is the first study focusing on whole blood lncH19 RNA expression in acromegaly patients. According to the obtained results, the whole blood expression of H19 is not a relevant diagnostic or monitoring marker for acromegaly. H19 is an intriguing biomarker for malignancies; however, we did not find advantages for its use for patients with benign pituitary tumours. Due to the small sizes of the groups, especially of de novo patients, which is a limitation of our study, these are preliminary results, and further investigations should be continued with larger groups. Additionally, association between tissue-specific and circulating H19 expression is a subject that should be explored. Acromegaly patients are at a higher risk of comorbid diseases, especially hypertension, dyslipidaemia, and goitre. For the first time, an association between cholelithiasis and H19 expression in acromegaly was revealed.

## Figures and Tables

**Table 1:** General characteristics of acromegaly patients and the control group.

	Acromegaly (N = 32)			Controls (N = 25)			
	Mean ± SD	Median	IQR	Mean ± SD	Median	IQR	*p*
**Sex (F%/M%)**	75/25			64/36			0.368
**Age (years)**	51.97 ± 13.93	48.00	43.00–64.50	41.08 ± 13.79	39.00	30.00–49.00	0.005
**Height (cm)**	165.11 ± 8.51	164.00	158.5–170.5	170.17 ± 9.05	168.00	164.00–180.00	0.045
**Weight (kg)**	84.43 ± 18.74	83.50	70.50–99.50	71.63 ± 18.36	69.00	57.00–82.00	0.018
**BMI (m^2^/kg)**	30.92 ± 6.42	31.05	26.37–35.27	24.55 ± 5.14	24.54	19.37–29.39	0.001
**IGF-I (ng/mL)**	275.31 ± 199.08	181.50	131.00–395.00	136.28 ± 54.29	138.00	93.70–153.00	0.004
**IGF-I/ULN**	1.12 ± 0.78	0.78	0.65–1.39	0.49 ± 0.16	0.50 ± 0.16	0.37–0.58	0.000

**Table 2:** H19 expression in acromegaly and control groups taking into account age (nonparametric ANCOVA in R with the WRS2 package).

	Age	n1	n2	Difference	SE	Lower CI Limit	Upper CI Limit	Test Value	*p*-Value
−ΔΔCt H19-BACT									
	37	18	18	0.00000	0.00030	−0.00080	0.00080	0.06	0.955
	39	16	19	0.00010	0.00030	−0.00070	0.00090	0.30	0.764
	45	19	14	−0.00010	0.00030	−0.00100	0.00090	0.23	0.819
	47	19	13	0.00010	0.00030	−0.00100	0.00110	0.15	0.882
	50	21	12	0.00010	0.00040	−0.00110	0.00130	0.32	0.756
−ΔΔCt H19-GAPDH									
	37	18	18	0.00030	0.00030	−0.00050	0.00120	1.20	0.244
	39	16	19	0.00040	0.00030	−0.00040	0.00120	1.42	0.175
	45	19	14	0.00020	0.00030	−0.00080	0.00110	0.49	0.630
	47	19	13	0.00020	0.00040	−0.00080	0.00120	0.55	0.589
	50	21	12	0.00000	0.00040	−0.00100	0.00110	0.06	0.950

**Table 3:** Comorbidities in acromegaly and the controls.

	Acromegaly (N/%)	Controls (N/%)	*p*
Hypertension	16 (50.0)	5 (20.0)	0.020
Dyslipidemia	21 (65.6)	10 (40.0)	0.054
Diabetes	9 (28.1)	2 (8.0)	0.090
Prediabetes	8 (25.0)	1 (4.0)	0.063
Goiter	21 (65.6)	3 (12.0)	0.000
Nephrolithiasis	3 (9.4)	0	0.248
Cholelithiasis	12 (37.5)	0	0.001
Colon polyps	3 (9.4)	0	0.248
Osteoporosis	2 (6.25)	1 (4.0)	1.000
Pituitary insufficiency	14 (43.75)	6 (24.0)	0.121

## Data Availability

The original contributions presented in the study are included in the article, further inquiries can be directed to the corresponding author.

## Supplementary Materials

The following supporting information can be downloaded at: https://www.mdpi.com/article/10.3390/biomedicines11041211/s1, Table S1: Difference in H19 expression between acromegaly (1) and the control group (2); Mann-Whitney test; Table S2: Difference in H19 expression between patients without operation and radiotherapy (1), successfully cured patients (2) and the control group (3); Kruskal-Wallis test; Table S3: Difference in H19 expression between patients unoperated/ineffectively operated (1), successfully operated (2) and the controls (3); Kruskal-Wallis test; Table S4: Difference in H19 expression between patients with active acromegaly (1), controlled or cured acromegaly (2) and the control group (3); Kruskal-Wallis test; Table S5: Difference in H19 expression between patients de novo (1), SSA treated (2) and the control group (3); Kruskal-Wallis test; Table S6: Difference in H19 expression between patients de novo (1), treated with radiotherapy (2) and the control group (3); Kruskal-Wallis test.

## References

[1] Katznelson L., Laws E.R., Melmed S., Molitch M.E., Murad M.H., Utz A., Wass J.A.H. (2014). Acromegaly: An Endocrine Society Clinical Practice Guideline. J. Clin. Endocrinol. Metab..

[2] Inoshita N., Nishioka H. (2018). The 2017 WHO Classification of Pituitary Adenoma: Overview and Comments. Brain Tumor Pathol..

[3] Ho K., Fleseriu M., Kaiser U., Salvatori R., Brue T., Lopes M.B., Kunz P., Molitch M., Camper S.A., Gadelha M. (2021). Pituitary Neoplasm Nomenclature Workshop: Does Adenoma Stand the Test of Time?. J. Endocr. Soc..

[4] Ershadinia N., Tritos N.A. (2022). Diagnosis and Treatment of Acromegaly: An Update. Mayo Clin. Proc..

[5] Giustina A., Barkhoudarian G., Beckers A., Ben-Shlomo A., Biermasz N., Biller B. (2020). Multidisciplinary Management of Acromegaly: A Consensus. Rev. Endocr. Metab. Disord..

[6] Gadelha M.R., Kasuki L., Lim D.S.T., Fleseriu M. (2019). Systemic Complications of Acromegaly and the Impact of the Current Treatment Landscape: An Update. Endocr. Rev..

[7] Pivonello R., Auriemma R.S., Grasso L.F.S., Pivonello C., Simeoli C., Patalano R., Galdiero M., Colao A. (2017). Complications of Acromegaly: Cardiovascular, Respiratory and Metabolic Comorbidities. Pituitary.

[8] Abreu A., Tovar A.P., Castellanos R., Valenzuela A., Giraldo C.M.G., Pinedo A.C., Guerrero D.P., Barrera C.A.B., Franco H.I., Ribeiro-Oliveira A. (2016). Challenges in the Diagnosis and Management of Acromegaly: A Focus on Comorbidities. Pituitary.

[9] Ponting C.P., Oliver P.L., Reik W. (2009). Evolution and Functions of Long Noncoding RNAs. Cell.

[10] Zhang P., Wu W., Chen Q., Chen M. (2019). Non-Coding RNAs and Their Integrated Networks. J. Integr. Bioinform..

[11] Shi X., Sun M., Liu H., Yao Y., Song Y. (2013). Long Non-Coding RNAs: A New Frontier in the Study of Human Diseases. Cancer Lett..

[12] Pachnis V., Belayew A., Tilghman S.M. (1984). Locus Unlinked to Alpha-Fetoprotein under the Control of the Murine Raf and Rif Genes. Proc. Natl. Acad. Sci. USA.

[13] Yoshimura H., Matsuda Y., Yamamoto M., Kamiya S., Ishiwata T. (2018). Expression and Role of Long Non-Coding RNA H19 in Carcinogenesis. Front. Biosci. Landmark.

[14] Ariel I., Ayesh S., Perlman E., Pizov G., Tanos V., Schneider T., Erdmann V., Podeh D., Komitowski D., Quasem A. (1997). The Product of the Imprinted H19 Gene Is an Oncofetal RNA. Mol. Pathol..

[15] Bartolomei M.S., Zemel S., Tilghman S.M. (1991). Parental Imprinting of the Mouse H19 Gene. Nature.

[16] Hibi K., Nakamura H., Hirai A., Fujikake Y., Kasai Y., Akiyama S., Ito K., Takagi H. (1996). Loss of H19 Imprinting in Esophageal Cancer. Cancer Res..

[17] Kondo M., Suzuki H., Ueda R., Osada H., Takagi K., Takahashi T. (1995). Frequent Loss of Imprinting of the H19 Gene Is Often Associated with Its Overexpression in Human Lung Cancers. Oncogene.

[18] Wu B., Zhang Y., Yu Y., Zhong C., Lang Q., Liang Z., Lv C., Xu F., Tian Y. (2021). Long Noncoding RNA H19: A Novel Therapeutic Target Emerging in Oncology Via Regulating Oncogenic Signaling Pathways. Front. Cell. Dev. Biol..

[19] Li H., Yu B., Li J., Su L., Yan M., Zhu Z., Liu B. (2014). Overexpression of LncRNA H19 Enhances Carcinogenesis and Metastasis of Gastric Cancer. Oncotarget.

[20] Verkerk A.J., Ariel I., Dekker M.C., Schneider T., van Gurp R.J., de Groot N., Gillis A.J., Oosterhuis J.W., Hochberg A.A., Looijenga L.H. (1997). Unique Expression Patterns of H19 in Human Testicular Cancers of Different Etiology. Oncogene.

[21] Berteaux N., Lottin S., Monté D., Pinte S., Quatannens B., Coll J., Hondermarck H., Curgy J.J., Dugimont T., Adriaenssens E. (2005). H19 MRNA-like Noncoding RNA Promotes Breast Cancer Cell Proliferation through Positive Control by E2F1. J. Biol. Chem..

[22] Qian B., Wang D.M., Gu X.S., Zhou K., Wu J., Zhang C.Y., He X.Y. (2018). LncRNA H19 Serves as a CeRNA and Participates in Non-Small Cell Lung Cancer Development by Regulating MicroRNA-107. Eur. Rev. Med. Pharmacol. Sci..

[23] Moulton T., Crenshaw T., Hao Y., Moosikasuwan J., Lin N., Dembitzer F., Hensle T., Weiss L., McMorrow L., Loew T. (1994). Epigenetic Lesions at the H19 Locus in Wilms’ Tumour Patients. Nat. Genet..

[24] Gicquel C., Raffin-Sanson M.-L., Gaston V., Bertagna X., Plouin P.-F., Schlumberger M., Louvel A., Luton J.-P., le Bouc Y. (1997). Structural and Functional Abnormalities at 11p15 Are Associated with the Malignant Phenotype in Sporadic Adrenocortical Tumors: Study on a Series of 82 Tumors. J. Clin. Endocrinol. Metab..

[25] Shima H., Kida K., Adachi S., Yamada A., Sugae S., Narui K., Miyagi Y., Nishi M., Ryo A., Murata S. (2018). Lnc RNA H19 Is Associated with Poor Prognosis in Breast Cancer Patients and Promotes Cancer Stemness. Breast Cancer Res. Treat..

[26] Si H., Chen P., Li H., Wang X. (2019). Long Non-Coding RNA H19 Regulates Cell Growth and Metastasis via MiR-138 in Breast Cancer. Am. J. Transl. Res..

[27] Luo J., Li Q., Pan J., Li L., Fang L., Zhang Y. (2018). Expression Level of Long Noncoding RNA H19 in Plasma of Patients with Nonsmall Cell Lung Cancer and Its Clinical Significance. J. Cancer Res. Ther..

[28] Huang C., Cao L., Qiu L., Dai X., Ma L., Zhou Y., Li H., Gao M., Li W., Zhang Q. (2015). Upregulation of H19 Promotes Invasion and Induces Epithelial-to-Mesenchymal Transition in Esophageal Cancer. Oncol. Lett..

[29] Tsang W.P., Ng E.K., Ng S.S., Jin H., Yu J., Sung J.J., Kwok T.T. (2010). Oncofetal H19-Derived MiR-675 Regulates Tumor Suppressor RB in Human Colorectal Cancer. Carcinogenesis.

[30] Zhong M.E., Chen Y., Zhang G., Xu L., Ge W., Wu B. (2019). LncRNA H19 Regulates PI3K-Akt Signal Pathway by Functioning as a CeRNA and Predicts Poor Prognosis in Colorectal Cancer: Integrative Analysis of Dysregulated NcRNA-Associated CeRNA Network. Cancer Cell. Int..

[31] Ariel I., Lustig O., Schneider T., Pizov G., Sappir M., De-Groot N., Hochberg A. (1995). The Imprinted H19 Gene as a Tumor Marker in Bladder Carcinoma. Urology.

[32] Wang J., Yang K., Yuan W., Gao Z. (2018). Determination of Serum Exosomal H19 as a Noninvasive Biomarker for Bladder Cancer Diagnosis and Prognosis. Med. Sci. Monit..

[33] Zhang K., Luo Z., Zhang Y., Zhang L., Wu L., Liu L., Yang J., Song X., Liu J. (2016). Circulating LncRNA H19 in Plasma as a Novel Biomarker for Breast Cancer. Cancer Biomark..

[34] Zhong G., Wang K., Li J., Xiao S., Wei W., Liu J. (2020). Determination of Serum Exosomal H19 as a Noninvasive Biomarker for Breast Cancer Diagnosis. Onco Targets Ther..

[35] Zhang Y., Liu Y.T., Tang H., Xie W.Q., Yao H., Gu W.T., Zheng Y.Z., Shang H.B., Wang Y., Wei Y.X. (2019). Exosome-Transmitted LncRNA H19 Inhibits the Growth of Pituitary Adenoma. J. Clin. Endocrinol. Metab..

[36] Zhong L., Liu P., Fan J., Luo Y. (2021). Long Non-Coding RNA H19: Physiological Functions and Involvements in Central Nervous System Disorders. Neurochem. Int..

[37] Wu Z.R., Yan L., Liu Y.T., Cao L., Guo Y.H., Zhang Y., Yao H., Cai L., Shang H.B., Rui W.W. (2018). Inhibition of MTORC1 by LncRNA H19 via Disrupting 4E-BP1/Raptor Interaction in Pituitary Tumours. Nat. Commun..

[38] Wu Z., Zheng Y., Xie W., Li Q., Zhang Y., Ren B., Cai L., Cheng Y., Tang H., Su Z. (2020). The Long Noncoding RNA-H19/MiRNA-93a/ATG7 Axis Regulates the Sensitivity of Pituitary Adenomas to Dopamine Agonists. Mol. Cell. Endocrinol..

[39] Zhou L., Wan Y., Cheng Q., Shi B., Zhang L., Chen S. (2020). The Expression and Diagnostic Value of LncRNA H19 in the Blood of Patients with Osteoarthritis. Iran. J. Public Health.

[40] Chen S., Liu D., Zhou Z., Qin S. (2021). Role of Long Non-Coding RNA H19 in the Development of Osteoporosis. Mol. Med..

[41] Su W., Huo Q., Wu H., Wang L., Ding X., Liang L., Zhou L., Zhao Y., Dan J., Zhang H. (2021). The Function of LncRNA-H19 in Cardiac Hypertrophy. Cell. Biosci..

[42] Safaei S., Tahmasebi-Birgani M., Bijanzadeh M., Seyedian S.M. (2020). Increased Expression Level of Long Noncoding RNA H19 in Plasma of Patients with Myocardial Infarction. Int. J. Mol. Cell. Med..

[43] Alfaifi M., Verma A.K., Alshahrani M.Y., Joshi P.C., Alkhathami A.G., Ahmad I., Hakami A.R., Beg M.M.A. (2020). Assessment of Cell-Free Long Non-Coding RNA-H19 and MiRNA-29a, MiRNA-29b Expression and Severity of Diabetes. Diabetes Metab. Syndr. Obes..

[44] Fawzy M.S., Abdelghany A.A., Toraih E.A., Mohamed A.M. (2020). Circulating Long Noncoding RNAs H19 and GAS5 Are Associated with Type 2 Diabetes but Not with Diabetic Retinopathy: A Preliminary Study. Bosn. J. Basic Med. Sci..

[45] Bolanowski M., Ruchała M., Zgliczyński W., Kos-Kudła B., Hubalewska-Dydejczyk A., Lewiński A. (2019). Diagnostics and Treatment of Acromegaly—Updated Recommendations of the Polish Society of Endocrinology. Endokrynol. Pol..

[46] Mair P., Wilcox R. (2020). Robust Statistical Methods in R Using the WRS2 Package. Behav. Res. Methods.

[47] Yballe C.M., Vu T.H., Hoffman A.R. (1996). Imprinting and Expression of Insulin-like Growth Factor-II and H19 in Normal Breast Tissue and Breast Tumor. J. Clin. Endocrinol. Metab..

[48] Arita T., Ichikawa D., Konishi H., Komatsu S., Shiozaki A., Shoda K., Kawaguchi T., Hirajima S., Nagata H., Kubota T. (2013). Circulating Long Non-Coding RNAs in Plasma of Patients with Gastric Cancer. Anticancer Res..

[49] Yu B., Cui R., Lan Y., Zhang J., Liu B. (2021). Long Non-Coding RNA H19 as a Diagnostic Marker in Peripheral Blood of Patients with Sepsis. Am. J. Transl. Res..

[50] Hashemian F., Ghafouri-Fard S., Arsang-Jang S., Mirzajani S., Fallah H., Mehvari Habibabadi J., Sayad A., Taheri M. (2019). Epilepsy Is Associated with Dysregulation of Long Non-Coding RNAs in the Peripheral Blood. Front. Mol. Biosci..

[51] Tello-Flores V.A., Valladares-Salgado A., Ramírez-Vargas M.A., Cruz M., del-Moral-Hernández O., Cahua-Pablo J.Á., Ramírez M., Hernández-Sotelo D., Armenta-Solis A., Flores-Alfaro E. (2020). Altered Levels of MALAT1 and H19 Derived from Serum or Serum Exosomes Associated with Type-2 Diabetes. Noncoding RNA Res..

[52] Lu T., Yu C., Ni H., Liang W., Yan H., Jin W. (2018). Expression of the Long Non-Coding RNA H19 and MALAT-1 in Growth Hormone-Secreting Pituitary Adenomas and Its Relationship to Tumor Behavior. Int. J. Dev. Neurosci..

[53] Zhu Z., Song L., He J., Sun Y., Liu X., Zou X. (2015). Ectopic Expressed Long Non-Coding RNA H19 Contributes to Malignant Cell Behavior of Ovarian Cancer. Int. J. Clin. Exp. Pathol..

[54] Colao A., Ferone D., Marzullo P., Lombardi G. (2004). Systemic Complications of Acromegaly: Epidemiology, Pathogenesis, and Management. Endocr. Rev..

[55] Rolla M., Jawiarczyk-Przybyłowska A., Halupczok-Żyła J., Kałużny M., Konopka B.M., Błoniecka I., Zieliński G., Bolanowski M. (2021). Complications and Comorbidities of Acromegaly—Retrospective Study in Polish Center. Front. Endocrinol..

[56] Tirosh A., Shimon I. (2017). Complications of Acromegaly: Thyroid and Colon. Pituitary.

[57] Montini M., Gianola D., Pagani M.D., Pedroncelli A., Caldara R., Gherardi F., Bonelli M., Lancranjan I., Pagani G. (1994). Cholelithiasis and Acromegaly: Therapeutic Strategies. Clin. Endocrinol..

[58] Attanasio R., Mainolfi A., Grimaldi F., Cozzi R., Montini M., Carzaniga C., Grottoli S., Cortesi L., Albizzi M., Testa R.M. (2008). Somatostatin Analogs and Gallstones: A Retrospective Survey on a Large Series of Acromegalic Patients. J. Endocrinol. Investig..

[59] Gan X., Liu S., Liang K. (2020). MicroRNA-19b-3p Promotes Cell Proliferation and Osteogenic Differentiation of BMSCs by Interacting with LncRNA H19. BMC Med. Genet..

[60] Li Z., Hong Z., Zheng Y., Dong Y., He W., Yuan Y., Guo J. (2020). An Emerging Potential Therapeutic Target for Osteoporosis: LncRNA H19/MiR-29a-3p Axis. Eur. J. Histochem..

[61] Zhang Y., Huang W., Yuan Y., Li J., Wu J., Yu J., He Y., Wei Z., Zhang C. (2020). Long Non-Coding RNA H19 Promotes Colorectal Cancer Metastasis via Binding to HnRNPA2B1. J. Exp. Clin. Cancer Res..

[62] Ding D., Li C., Zhao T., Li D., Yang L., Zhang B. (2018). LncRNA H19/MiR-29b-3p/PGRN Axis Promoted Epithelial-Mesenchymal Transition of Colorectal Cancer Cells by Acting on Wnt Signaling. Mol. Cells.

