# Supplementary material for: Is H19 RNA a Useful Marker of Acromegaly and Its Complications? A Preliminary Study
Source: Biomedicines. 2023 Apr 19;11(4):1211. doi: 10.3390/biomedicines11041211 (PMC10136071; doi:10.3390/biomedicines11041211)
Supplement: Supplementary file 1 [file biomedicines-11-01211-s001.zip › biomedicines-2297001-supplementary.pdf]

Table S1: Difference in H19 expression between acromegaly (1) and the control group (2); Mann-Whitney test.

	n1	n2	p
-ΔΔCt H19-BACT	32	25	0.091
-ΔΔCt H19-GAPDH			0.259

Table S2: Difference in H19 expression between patients without operation and radiotherapy (1), successfully cured patients (2) and the control group (3); Kruskal-Wallis test.

	n1	n2	n3	p
-ΔΔCt H19-BACT	6	12	25	0.493
-ΔΔCt H19-GAPDH				0.792

Table S3: Difference in H19 expression between patients unoperated/ineffectively operated (1), successfully operated (2) and the controls (3); Kruskal-Wallis test.

	n1	n2	n3	p
-ΔΔCt H19-BACT	20	12	25	0.218
-ΔΔCt H19-GAPDH				0.349

Table S4: Difference in H19 expression between patients with active acromegaly (1), controlled or cured acromegaly (2) and the control group (3); Kruskal-Wallis test.

	n1	n2	n3	p
-ΔΔCt H19-BACT	11	21	25	0.23
-ΔΔCt H19-GAPDH				0.216

Table S5: Difference in H19 expression between patients de novo (1), SSA treated (2) and the control group (3); Kruskal-Wallis test.

	n1	n2	n3	p
-ΔΔCt H19-BACT	5	15	25	0.207
-ΔΔCt H19-GAPDH				0.353

Table S6: Difference in H19 expression between patients de novo (1), treated with radiotherapy (2) and the control group (3); Kruskal-Wallis test.

	n1	n2	n3	p

-ΔΔCt H19-BACT	5	4	25	0.122
-ΔΔCt H19-GAPDH				0.579
